# Static and Dynamic Changes of Amplitude of Low-Frequency Fluctuations in Cervical Discogenic Pain

**DOI:** 10.3389/fnins.2020.00733

**Published:** 2020-07-14

**Authors:** Mingyue Ma, Hong Zhang, Run Liu, Hongsheng Liu, Xiangchun Yang, Xiaohui Yin, Song Chen, Xiaoping Wu

**Affiliations:** ^1^Department of Radiology, The Affiliated Xi’an Central Hospital of Xi’an Jiaotong University, Xi’an, China; ^2^Department of Radiology, The Affiliated Xi’an XD Group Hospital of Shanxi University of Traditional Chinese Medicine, Xi’an, China

**Keywords:** cervical discogenic pain, ALFF, resting-state, static, dynamic

## Abstract

Cervical discogenic pain (CDP) is a clinically common pain syndrome caused by cervical disk degeneration. A large number of studies have reported that CDP results in brain functional impairments. However, the detailed dynamic brain functional abnormalities in CDP are still unclear. In this study, using resting-state functional magnetic resonance imaging, we explored the neural basis of CDP with 40 CDP patients and 40 age-, gender-matched healthy controls to delineate the changes of the voxel-level static and dynamic amplitude of low frequency fluctuations (ALFF). We found increased static ALFF in left insula (INS) and posterior precuneus (PCu), and decreased static ALFF in left precentral/postcentral gyrus (PreCG/PoCG), thalamus (THA), and subgenual anterior cingulate cortex in CPD patients compared to healthy controls. We also found decreased dynamic ALFF in left PreCG/PoCG, right posterior middle temporal gyrus, and bilateral THA. Moreover, we found that static ALFF in left PreCG/PoCG and dynamic ALFF in THA were significantly negatively correlated with visual analog scale and disease duration, respectively. Our findings provide the neurophysiological basis for CDP and facilitate understanding the neuropathology of CDP.

## Introduction

Cervical discogenic pain (CDP) is a clinically common pain syndrome caused by cervical disk degeneration which results in chronic pain in the head, neck, shoulder and upper limbs, as well as pain associated with numbness (Tracy and Bartleson, 2010; Thoomes et al., 2012). Long-term chronic pain seriously affects patients’ physical and mental health and quality of life. The clinical studies have demonstrated that patients with long-term duration chronic pain led to brain functional impairments of sensory, motor, cognition, memory, and affective processing (Montero-Homs, 2009; Linton, 2013; Denkinger et al., 2014). Although a few studies have reported functional connectivity and local regional homogeneity abnormalities in CDP patients using resting-state functional magnetic resonance imaging (fMRI) (Yu et al., 2017; Woodworth et al., 2018), the dynamic changes of the low-frequency oscillation in CDP is remains unknown.

Resting-state fMRI is commonly used to measure the spontaneous low-frequency fluctuations of brain activities with blood oxygen level-dependent (BOLD) signals (Biswal et al., 1995; Fox and Raichle, 2007). A large number of previous studies applied resting-state fMRI to reveal the intrinsic functional organization of brain in healthy subjects and to identify the changes of the intrinsic functional connectivities under diseases (Greicius et al., 2003; Fox et al., 2006; Wang et al., 2015, 2017d; Sun et al., 2018; Xu et al., 2019a; Gao et al., 2020). Moreover, many recent studies have applied resting-state fMRI to reveal the brain mechanism of neuromodulation treatment for clinical disorders (Peng et al., 2012; Liu et al., 2015; Wang et al., 2017b,c,d, 2020; Wang J. et al., 2018; Bai et al., 2018, 2019). The amplitude of low frequency fluctuations (ALFF) is a main measurement to characterize the functional activity during resting-sate (Zang et al., 2007). Combining the ALFF with sliding-window approach, the dynamic ALFF (dALFF) method was proposed to measure the variance of ALFF over time (Fu et al., 2018). The dALFF provides a new avenue to depict time-varying local brain activity. Generally, the static ALFF was calculated by taking all the time points’ series as a whole for power analyses, whereas the dALFF was calculated by dividing the whole time series into different small segmentations for power analysis. Therefore, using static and dynamic ALFF may provide a complementary evidence to uncover the brain functional changes in patients with CDP.

In this study, we aimed to identify the brain functional abnormalities caused by CDP using static and dynamic ALFF method in 40 CDP patients and 40 age- and gender-matched healthy controls. We hypothesized that the static and dynamic could reveal different but overlapped brain areas showing functional abnormalities. The voxel-wise static and dynamic ALFF maps were calculated and compared between CDP patients and healthy controls. The correlation analyses were performed to identify the relationships between ALFF measurements and clinical characteristics.

## Materials and Methods

### Participants

Forty right-handed CDP patients (22 females and 18 males; mean age: 53.6 ± 6.9 years) and 40 gender- and age-matched healthy controls (22 females and 18 males; mean age: 52.8 ± 7.6 years) were used in current study. All the CDP patients were diagnosed by one orthopedist with physical and imaging examination. A main characteristic for CDP is that the damaged disk showed reduced weighted T2 signals compared to the neighboring normal disk. The patients with cervical disk herniation, disk degeneration and stenosis, and cervical spondylosis were excluded. Moreover, only the pain caused by the internal disorder of the intervertebral disk not by radiation pain or segmental nerve disorder was considered to be CDP. The CDP patients with hypertension, diabetes, mental illness, intracranial infection, craniocerebral trauma, MRI contraindication, and a history of chronic pain were excluded. Forty right-handed healthy controls were recruited from the community and not have any hypertension, diabetes, mental illness, intracranial infection, craniocerebral trauma, and a history of chronic pain. The clinical measures were obtained to assess the pain intensity, cognitive functions and emotional states. The visual analog scale (VAS) test was used to assess the pain intensity. The Montreal cognitive assessment scale (MoCA) was used to evaluate the cognitive ability. The Hamilton depression scale (HAMD) and Hamilton anxiety scale (HAMA) were applied to determine their depression and anxiety levels, respectively. The written informed consents were provided and obtained from all subjects. The study was approved by the Ethics Committee of the Affiliated Xi’an Central Hospital of Xi’an Jiaotong University and was accordance with the Helsinki declaration and its later amendments or comparable ethical standards.

### Resting-State fMRI Data Acquisition

The resting-state fMRI data was collected using a Philips 3.0T MRI scanner. All the subjects were asked not to move as much as possible and to keep their eyes closed without falling asleep. Foam pads and headphones were used to reduce head movement and scanner noise. The resting-state fMRI data was collected using echo plane imaging (EPI) with the following parameters: repetition time/echo time ratio = 2000/30 ms, flip angle = 90°, acquisition matrix size = 64 × 64, field of view = 230 × 230, the voxel size = 3.6 mm × 3.6 mm × 3.6 mm, 38 slices with 0.6 mm gap, 240 volumes.

### Resting-State fMRI Data Preprocessing

The resting-state fMRI data was preprocessed using the toolkit of DPARSF version 2.3.^1^ The first 10 volumes were discarded to facilitate magnetization equilibrium effects and were realigned to the first volume to correct head motion. Then, the fMRI images were normalized to EPI template and resampled to 3 mm^3^ isotropic voxels. Friston 24-parameter of head motion, white matter, cerebrospinal fluid, and whole brain mean signals were regressed out and the fMRI images were filtered a frequency band of 0.01–0.08 Hz. Finally, the voxel-wise static ALFF and dynamic ALFF were calculated.

### Static and Dynamic ALFF Calculation

The ALFF was mainly used to characterize the resting-state functional activity of each brain voxel (Zou et al., 2008). To calculate the static ALFF, the time series of each voxel was first transformed to frequency domain for power spectrum calculation. ALFF is computed at the power within the low-frequency range (0.01–0.1 Hz). The dynamic ALFF was calculated using a sliding window method. For dynamic ALFF calculation, the length of sliding window is absence of a standard criterion. To exclude the spurious fluctuations, many previous studies have demonstrated the minimum window length should be larger than 1/*f*_min_, where *f*_min_ is the minimum frequency of time series (Leonardi and Van De Ville, 2015; Du et al., 2017; Li et al., 2019). Thus, a window length of 50 TR (100 s) with step size of 5 TR (10 s) as the optimal parameter was applied in this study to keep the balance between capturing a reliable dynamics and to obtain steady correlations between regions for dynamic ALFF calculation. In each window, the ALFF map was computed, and the variance of the ALFF maps across all the windows was used to measure the dynamic. Finally, the static and dynamic ALFF maps were normalized to z-scores and smoothed with 6 mm Gaussian kernel for statistical analyses.

### Statistical and Correlation Analyses

Two-sample *t*-tests with MoCA, HAMD, and HAMA as covariates were performed to compare the static and dynamic ALFF maps between healthy controls and CDP patients. The significance was determined using a Gaussian random field (GRF) correction method with *p* < 0.05.

To explore the relationship between the static and dynamic ALFF values and clinical measurements, correlation analyses were performed between the regional mean static and dynamic ALFF values and VAS, MoCA, HAMD, and HAMA scores in CDP patients. The significance was set at *p* < 0.05 with Bonferroni correction.

## Results

### Clinical Characteristics

There were no significant differences in age (*p* = 0.15) between CDP patients and healthy controls (Table 1). There were significantly decreased MoCA and increased HAMD, HAMA scores in CDP patients compared to healthy controls (Table 1).

**TABLE 1:** Demographics characteristics of cervical discogenic pain (CDP) and healthy controls (HC).

**Characteristic**	**CDP**	**HC**	***p*-value**
Sample size (F/M)	40 (22/18)	40 (22/18)	NS
Age (Mean ± SD years)	53.6 ± 6.9	52.8 ± 7.6	0.78
VAS	6.73 ± 1.65		NA
MoCA	17.03 ±1.83	26.38 ± 3.67	<0.001
HAMD	4.94 ± 3.95	0.91 ± 0.73	<0.001
HAMA	5.27 ± 3.78	0.77 ± 0.59	<0.001
Duration of pain, year	3.25 ± 1.46		NA

*F, female; M, male; VAS, visual analog scale; MoCA, Montreal Cognitive Assessment; HAMD, Hamilton Depression Rating Scale; HAMA, Hamilton Anxiety Scale; NS, not significant; NA, not available.*

### Changed Static ALFF

**FIGURE 1:**
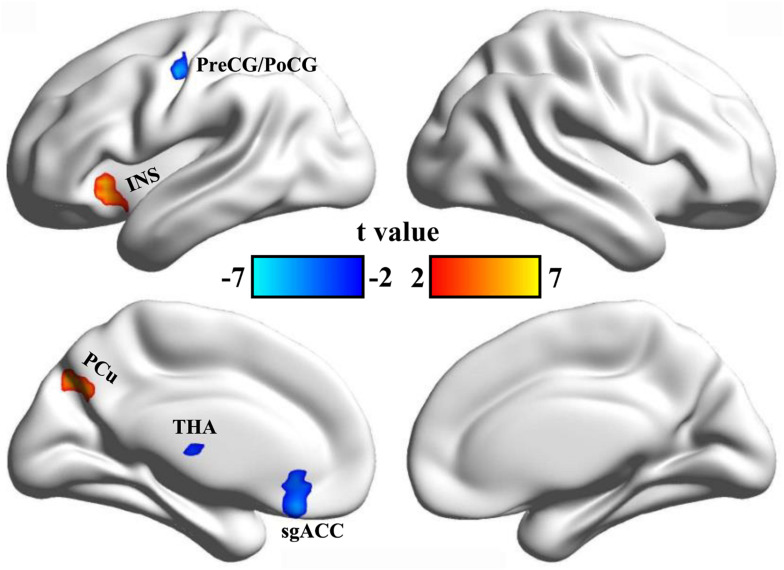
Changed static ALFF in CDP patients. A two-sample *t*-test was used to compare the static ALFF maps between CDP patients and healthy controls. Significantly increased static ALFF in left anterior insula and left posterior precuneus were found in CDP patients. Moreover, significant reeducations of static ALFF in left precentral/postcentral gyrus, subgenual anterior cingulate cortex, and thalamus were found in CDP patients.

Static ALFF analyses identified increased ALFF in the left insula (INS) and the left precuneus (PCu), and decreased ALFF in left precentral/postcentral gyrus (PreCG/PoCG), thalamus (THA), and subgenual anterior cingulate cortex (sgACC) in CDP patients compared to healthy controls (Table 2 and Figure 1).

**TABLE 2:** Regions with changed static and dynamic ALFF in CDP patients.

**Indices**	**Brain Regions**	**L/R**	**Peak MNI Coordinates**			***t-*values**
			**X**	**Y**	**Z**	
Static ALFF:	Anterior insula	L	−36	24	−3	7.2
	Precuneus	L	−18	−72	39	6.0
	Subgenual anterior cingulate cortex	L	−6	30	−18	−5.3
	Thalamus	L	−6	−18	6	−7.6
	Precentral/postcentral gyrus	L	−42	−9	45	−5.9
dynamic ALFF:	Posterior middle temporal gyrus	R	54	−54	−3	−7.5
	Thalamus	L	−12	3	3	−6.0
	Precentral/postcentral gyrus	L	−48	−9	33	−5.6

*CDP, cervical discogenic pain; MNI, Montreal Neurological Institute; ALFF, amplitude of low frequency fluctuations; L, left hemisphere; R, right hemisphere.*

### Changed Dynamic ALFF

**FIGURE 2:**
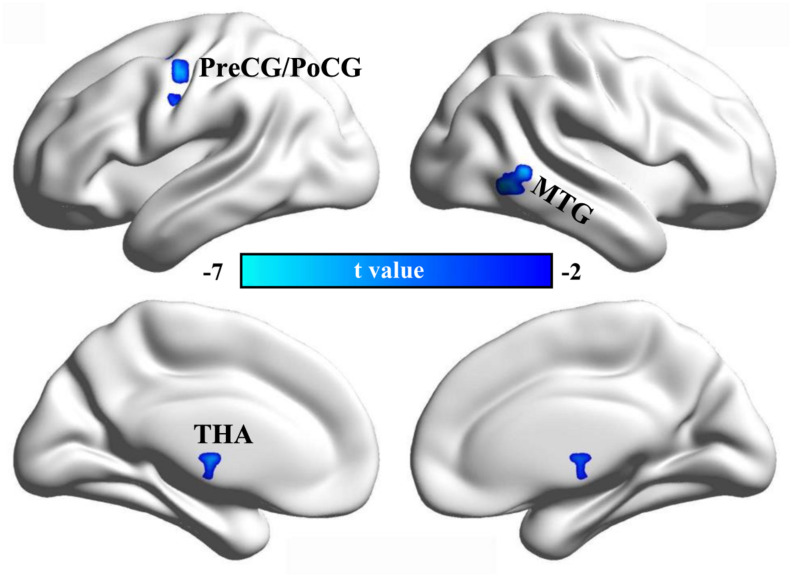
Changed dynamic ALFF in CDP patients. A two-sample *t*-test was used to compare the dynamic ALFF maps between CDP patients and healthy controls. Significantly decreased dynamic ALFF in left precentral/postcentral gyrus, right posterior middle temporal gyrus, and bilateral thalamus were found in CDP patients.

Using dynamic ALFF analysis, we found decreased dynamic ALFF in the left PreCG/PoCG, the right posterior middle temporal gyrus (MTG), and the bilateral THA in CDP patients compared to healthy controls (Table 2 and Figure 2).

### Correlation Analyses

**FIGURE 3:**
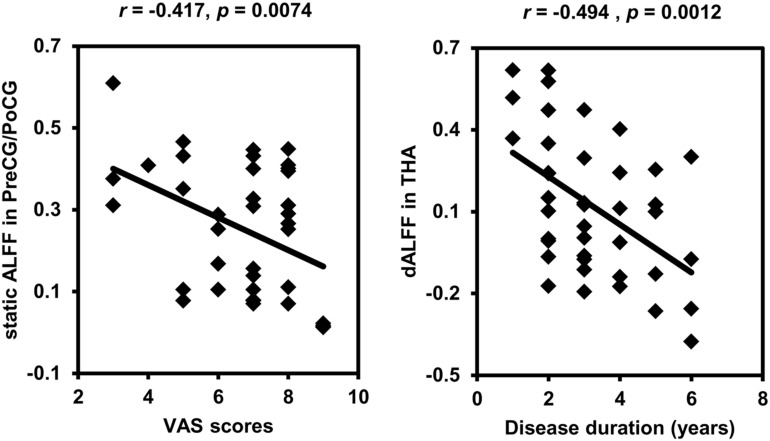
Correlation analyses between static, dynamic ALFF and clinical characteristics. Significant correlations were found between the static ALFF in left precentral/postcentral gyrus and VAS scores and between the dynamic ALFF in left thalamus and disease duration in CDP patients.

Correlation analyses identified negative correlations between static ALFF in left PreCG/PoCG and VAS scores (*r* = −0.417, *p* = 0.0074), and between dynamic ALFF in left THA and disease duration (*r* = −0.494, *p* = 0.0012) in CDP patients (Figure 3).

## Discussion

In this study, we identified changed static and dynamic ALFF in insula, precentral/postcentral gyrus, precuneus, posterior middle temporal gyrus, and bilateral thalamus in CDP patients. Moreover, the static ALFF in precentral/postcentral gyrus and dynamic ALFF in thalamus were negatively correlated with VAS scores and duration of disease, respectively. Our findings revealed dynamic changes of brain functional activities in CDP patients which provide the neurophysiological basis for CDP.

We found increased static ALFF in left insula and precuneus in CDP patients. The insula is a heterogeneous area and is important for processing of emotion and pain perception (Dosenbach et al., 2007; Thielscher and Pessoa, 2007; Kelly et al., 2012). The insula is a key part of the pain matrix and trigger the pain matrix network for subjective pain experience (Taylor et al., 2009; Isnard et al., 2011; Favilla et al., 2014). The insula is also critical for emotional processing and pain memory (Gasquoine, 2014). A large number of studies have revealed the functional disruption of anterior insula in depression and pain-related diseases (Wang et al., 2017a; Wang J. et al., 2020; Wang Y. et al., 2018, 2020; Wang L. et al., 2020). The posterior precuneus mainly participates in visual and memory processing (Zhang et al., 2014; Wang et al., 2019). Thus, increased ALFF in anterior insula and posterior precuneus may be a compensatory mechanism and is related to elevated pain memory and abnormal emotion and visuospatial attention processing in CDP patients.

We also found decreased static and dynamic ALFF in sensorimotor network including precentral/postcentral gyrus and bilateral thalamus (Otti et al., 2013). This finding was supported by previous studies in cervical spondylosis and chronic knee osteoarthritis (Liao et al., 2018; Woodworth et al., 2019). The sensorimotor network is involved in processing changing pain intensity and participates in discrimination of the sensory components of pain perception (Kanda et al., 2000; Peyron et al., 2000). The pain intensity processing of SMN network was supported by our correlation analysis between the static ALFF in precentral/postcentral gyrus and VAS scores. The thalamus is a key gate for processing sensory and motor signals and is as a hub for information exchanges between different subcortical areas and the cerebral cortex (Hwang et al., 2017; Lipiec and Wisniewska, 2019; Tang et al., 2020). It is also plays an important role in regulation of consciousness (Steriade and Llinas, 1988; Redinbaugh et al., 2020). The important role of thalamus in pain processing was further supported by the correlation analysis of the dynamic ALFF and disease duration in our study. In a word, all these findings demonstrated the disrupted low frequency oscillation of SMN network may be a basic neuropathology of CDP.

Moreover, we found decreased static ALFF in left subgenual anterior cingulate cortex and decreased dynamic ALFF in right posterior MTG in CDP patients. The posterior MTG has been reported to be involved in social cognitive and language processing (Xu et al., 2015, 2019a). The disrupted functional connectivity pattern of the posterior MTG is closely associated the autism spectrum disorder (Xu et al., 2019b). The sgACC is a key region for emotion, reward and decision making processing (Johansen-Berg et al., 2008), and the structural abnormality has been identified in patients with chronic knee osteoarthritis pain (Liao et al., 2018). Functional impairments of sgACC has also been widely observed in depression (Drevets et al., 1998, 2008; Botteron et al., 2002; Wu et al., 2016). Therefore, the decreased ALFF in the two areas may suggest the abnormal reward, emotion, and social cognition processing in CDP patients.

This study has several limitations. First, the selection of the sliding window length remains a topic of debate, and optimal length for obtaining the dynamics of brain activity is unclear. We selected 50 TR as window length on the basis of the criteria that the minimum length should be larger than 1/f min, which was proposed by previous studies (Leonardi and Van De Ville, 2015; Li et al., 2019). The results of different sliding window lengths were similar to the main results of 50 TR, demonstrating that our findings of dALFF variability were relatively stable. Second, given the high comorbidity of anxiety and depression, excluding individuals with depressive disorder may decrease the generalizability of our findings. More comorbid samples are required to replicate and complement our findings.

There are several limitations. First, how to select the length of sliding window is still an open problem, which is a main drawback of dynamic method. Second, all the patients took medications, which may affect the current findings. Third, the samples is still small, the future studies with a larger samples are needed.

## Conclusion

Our current study revealed the static and dynamic changes of low-frequency oscillation in CDP patients. We identified increased ALFF in insula and posterior precuneus which may reflect the elevated pain memory. Moreover, we found the decreased ALFF in sensorimotor, social cognition and emotional networks indicating that chronic pain will result functional impairments in emotion, sensorimotor and social cognition. Our findings further demonstrated that combing static and dynamic analyses can provide complementary evidence for understand the neuropathology of CDP.

## Data Availability Statement

The raw data supporting the conclusions of this article will be made available by the authors, without undue reservation.

## Ethics Statement

The studies involving human participants were reviewed and approved by the Affiliated Xi’an Central Hospital of Xi’an Jiaotong University. The patients/participants provided their written informed consent to participate in this study.

## Author Contributions

All authors listed have made a substantial, direct and intellectual contribution to the work, and approved it for publication.

## Conflict of Interest

The authors declare that the research was conducted in the absence of any commercial or financial relationships that could be construed as a potential conflict of interest.

## Footnotes

1 www.restfmri.net/forum/DPARSF

## References

[B1] Bai T. Wei Q. Xie W. Wang A. Wang J. Ji G. J. (2018). Hippocampal-subregion functional alterations associated with antidepressant effects and cognitive impairments of electroconvulsive therapy. *Psychol. Med.* 49 1357–1364. 10.1017/s0033291718002684 30229715 PMC6518386

[B2] Bai T. Wei Q. Zu M. Xie W. Wang J. Gong-Jun J. (2019). Functional plasticity of the dorsomedial prefrontal cortex in depression reorganized by electroconvulsive therapy: validation in two independent samples. *Hum. Brain Mapp.* 40 465–473. 10.1002/hbm.24387 30240504 PMC6865625

[B3] Biswal B. Yetkin F. Z. Haughton V. M. Hyde J. S. (1995). Functional connectivity in the motor cortex of resting human brain using echo-planar MRI. *Magn. Reson. Med.* 34 537–541. 10.1002/mrm.1910340409 8524021

[B4] Botteron K. N. Raichle M. E. Drevets W. C. Heath A. C. Todd R. D. (2002). Volumetric reduction in left subgenual prefrontal cortex in early onset depression. *Biol. Psychiatry* 51 342–344. 10.1016/s0006-3223(01)01280-x 11958786

[B5] Denkinger M. D. Lukas A. Nikolaus T. Peter R. Franke S. (2014). Multisite pain, pain frequency and pain severity are associated with depression in older adults: results from the ActiFE Ulm study. *Age Ageing* 43 510–514. 10.1093/ageing/afu013 24603284

[B6] Dosenbach N. U. Fair D. A. Miezin F. M. Cohen A. L. Wenger K. K. Dosenbach R. A. (2007). Distinct brain networks for adaptive and stable task control in humans. *Proc. Natl. Acad. Sci. U.S.A.* 104 11073–11078. 10.1073/pnas.0704320104 17576922 PMC1904171

[B7] Drevets W. C. Ongur D. Price J. L. (1998). Neuroimaging abnormalities in the subgenual prefrontal cortex: implications for the pathophysiology of familial mood disorders. *Mol. Psychiatry* 3 190–221. 9672897 10.1038/sj.mp.4000370

[B8] Drevets W. C. Savitz J. Trimble M. (2008). The subgenual anterior cingulate cortex in mood disorders. *CNS Spectr.* 13 663–681. 10.1017/s1092852900013754 18704022 PMC2729429

[B9] Du Y. Pearlson G. D. Lin D. Sui J. Chen J. Salman M. (2017). Identifying dynamic functional connectivity biomarkers using GIG-ICA: application to schizophrenia, schizoaffective disorder, and psychotic bipolar disorder. *Hum. Brain Mapp.* 38 2683–2708. 10.1002/hbm.23553 28294459 PMC5399898

[B10] Favilla S. Huber A. Pagnoni G. Lui F. Facchin P. Cocchi M. (2014). Ranking brain areas encoding the perceived level of pain from fMRI data. *Neuroimage* 90 153–162. 10.1016/j.neuroimage.2014.01.001 24418504

[B11] Fox M. D. Corbetta M. Snyder A. Z. Vincent J. L. Raichle M. E. (2006). Spontaneous neuronal activity distinguishes human dorsal and ventral attention systems. *Proc. Natl. Acad. Sci. U.S.A.* 103 10046–10051. 10.1073/pnas.0604187103 16788060 PMC1480402

[B12] Fox M. D. Raichle M. E. (2007). Spontaneous fluctuations in brain activity observed with functional magnetic resonance imaging. *Nat. Rev. Neurosci.* 8 700–711. 10.1038/nrn2201 17704812

[B13] Fu Z. Tu Y. Di X. Du Y. Pearlson G. D. Turner J. A. (2018). Characterizing dynamic amplitude of low-frequency fluctuation and its relationship with dynamic functional connectivity: an application to schizophrenia. *Neuroimage* 180 619–631. 10.1016/j.neuroimage.2017.09.035 28939432 PMC5860934

[B14] Gao Z. Guo X. Liu C. Mo Y. Wang J. (2020). Right inferior frontal gyrus: an integrative hub in tonal bilinguals. *Hum. Brain Mapp.* 41 2152–2159. 10.1002/hbm.24936 31957933 PMC7268011

[B15] Gasquoine P. G. (2014). Contributions of the insula to cognition and emotion. *Neuropsychol. Rev.* 24 77–87. 10.1007/s11065-014-9246-9 24442602

[B16] Greicius M. D. Krasnow B. Reiss A. L. Menon V. (2003). Functional connectivity in the resting brain: a network analysis of the default mode hypothesis. *Proc. Natl. Acad. Sci. U.S.A.* 100 253–258. 10.1073/pnas.0135058100 12506194 PMC140943

[B17] Hwang K. Bertolero M. A. Liu W. B. D’Esposito M. (2017). The human thalamus is an integrative hub for functional brain networks. *J. Neurosci.* 37 5594–5607. 10.1523/jneurosci.0067-17.2017 28450543 PMC5469300

[B18] Isnard J. Magnin M. Jung J. Mauguiere F. Garcia-Larrea L. (2011). Does the insula tell our brain that we are in pain? *Pain* 152 946–951. 10.1016/j.pain.2010.12.025 21277680

[B19] Johansen-Berg H. Gutman D. A. Behrens T. E. Matthews P. M. Rushworth M. F. Katz E. (2008). Anatomical connectivity of the subgenual cingulate region targeted with deep brain stimulation for treatment-resistant depression. *Cereb. Cortex* 18 1374–1383. 10.1093/cercor/bhm167 17928332 PMC7610815

[B20] Kanda M. Nagamine T. Ikeda A. Ohara S. Kunieda T. Fujiwara N. (2000). Primary somatosensory cortex is actively involved in pain processing in human. *Brain Res.* 853 282–289. 10.1016/s0006-8993(99)02274-x 10640625

[B21] Kelly C. Toro R. Di Martino A. Cox C. L. Bellec P. Castellanos F. X. (2012). A convergent functional architecture of the insula emerges across imaging modalities. *Neuroimage* 61 1129–1142. 10.1016/j.neuroimage.2012.03.021 22440648 PMC3376229

[B22] Leonardi N. Van De Ville D. (2015). On spurious and real fluctuations of dynamic functional connectivity during rest. *Neuroimage* 104 430–436. 10.1016/j.neuroimage.2014.09.007 25234118

[B23] Li C. Xia L. Ma J. Li S. Liang S. Ma X. (2019). Dynamic functional abnormalities in generalized anxiety disorders and their increased network segregation of a hyperarousal brain state modulated by insomnia. *J. Affect. Disord.* 246 338–345. 10.1016/j.jad.2018.12.079 30597294

[B24] Liao X. Mao C. Wang Y. Zhang Q. Cao D. Seminowicz D. A. (2018). Brain gray matter alterations in Chinese patients with chronic knee osteoarthritis pain based on voxel-based morphometry. *Medicine* 97:e0145. 10.1097/md.0000000000010145 29561420 PMC5895331

[B25] Linton S. J. (2013). A transdiagnostic approach to pain and emotion. *J. Appl. Biobehav. Res.* 18 82–103. 10.1111/jabr.12007 24143062 PMC3796860

[B26] Lipiec M. A. Wisniewska M. B. (2019). We have to find a way - growth and guidance of thalamocortical axons. *Postepy Biochem.* 65 135–142. 31642652 10.18388/pb.2019_248

[B27] Liu C. Dai Z. Zhang R. Zhang M. Hou Y. Qi Z. (2015). Mapping intrinsic functional brain changes and repetitive transcranial magnetic stimulation neuromodulation in idiopathic restless legs syndrome: a resting-state functional magnetic resonance imaging study. *Sleep Med.* 16 785–791. 10.1016/j.sleep.2014.12.029 25959094

[B28] Montero-Homs J. (2009). [Nocioceptive pain, neuropathic pain and pain memory]. *Neurologia* 24 419–422. 19798608

[B29] Otti A. Guendel H. Henningsen P. Zimmer C. Wohlschlaeger A. M. Noll-Hussong M. (2013). Functional network connectivity of pain-related resting state networks in somatoform pain disorder: an exploratory fMRI study. *J. Psychiatry Neurosci.* 38 57–65. 10.1503/jpn.110187 22894821 PMC3529220

[B30] Peng H. Zheng H. Li L. Liu J. Zhang Y. Shan B. (2012). High-frequency rTMS treatment increases white matter FA in the left middle frontal gyrus in young patients with treatment-resistant depression. *J. Affect. Disord.* 136 249–257. 10.1016/j.jad.2011.12.006 22217432

[B31] Peyron R. Laurent B. Garcia-Larrea L. (2000). Functional imaging of brain responses to pain. A review and meta-analysis. *Neurophysiol. Clin.* 30 263–288. 10.1016/s0987-7053(00)00227-6 11126640

[B32] Redinbaugh M. J. Phillips J. M. Kambi N. A. Mohanta S. Andryk S. Dooley G. L. (2020). Thalamus modulates consciousness via layer-specific control of cortex. *Neuron* 106 66–75.e12. 10.1016/j.neuron.2020.01.005 32053769 PMC7243351

[B33] Steriade M. Llinas R. R. (1988). The functional states of the thalamus and the associated neuronal interplay. *Physiol. Rev.* 68 649–742. 10.1152/physrev.1988.68.3.649 2839857

[B34] Sun H. Luo L. Yuan X. Zhang L. He Y. Yao S. (2018). Regional homogeneity and functional connectivity patterns in major depressive disorder, cognitive vulnerability to depression and healthy subjects. *J. Affect. Disord.* 235 229–235. 10.1016/j.jad.2018.04.061 29660636

[B35] Tang Y. Wang M. Zheng T. Yuan F. Yang H. Han F. (2020). Grey matter volume alterations in trigeminal neuralgia: a systematic review and meta-analysis of voxel-based morphometry studies. *Prog. Neuropsychopharmacol. Biol. Psychiatry* 98:109821. 10.1016/j.pnpbp.2019.109821 31756417

[B36] Taylor K. S. Seminowicz D. A. Davis K. D. (2009). Two systems of resting state connectivity between the insula and cingulate cortex. *Hum. Brain Mapp.* 30 2731–2745. 10.1002/hbm.20705 19072897 PMC6871122

[B37] Thielscher A. Pessoa L. (2007). Neural correlates of perceptual choice and decision making during fear-disgust discrimination. *J. Neurosci.* 27 2908–2917. 10.1523/jneurosci.3024-06.2007 17360913 PMC6672587

[B38] Thoomes E. J. Scholten-Peeters G. G. de Boer A. J. Olsthoorn R. A. Verkerk K. Lin C. (2012). Lack of uniform diagnostic criteria for cervical radiculopathy in conservative intervention studies: a systematic review. *Eur. Spine J.* 21 1459–1470. 10.1007/s00586-012-2297-9 22531897 PMC3535232

[B39] Tracy J. A. Bartleson J. D. (2010). Cervical spondylotic myelopathy. *Neurologist* 16 176–187. 20445427 10.1097/NRL.0b013e3181da3a29

[B40] Wang C. Wu H. Chen F. Xu J. Li H. Li H. (2017a). Disrupted functional connectivity patterns of the insula subregions in drug-free major depressive disorder. *J. Affect. Disord.* 234 297–304. 10.1016/j.jad.2017.12.033 29587165

[B41] Wang J. Becker B. Wang L. Li H. Zhao X. Jiang T. (2019). Corresponding anatomical and coactivation architecture of the human precuneus showing similar connectivity patterns with macaques. *Neuroimage* 200 562–574. 10.1016/j.neuroimage.2019.07.001 31276799

[B42] Wang J. Wei Q. Bai T. Zhou X. Sun H. Becker B. (2017b). Electroconvulsive therapy selectively enhanced feedforward connectivity from fusiform face area to amygdala in major depressive disorder. *Soc. Cogn. Affect. Neurosci.* 12 1983–1992. 10.1093/scan/nsx100 28981882 PMC5716231

[B43] Wang J. Wei Q. Yuan X. Jiang X. Xu J. Zhou X. (2017c). Local functional connectivity density is closely associated with the response of electroconvulsive therapy in major depressive disorder. *J. Affect. Disord.* 225 658–664. 10.1016/j.jad.2017.09.001 28910748

[B44] Wang J. Xie S. Guo X. Becker B. Fox P. T. Eickhoff S. B. (2017d). Correspondent functional topography of the human left inferior parietal lobule at rest and under task revealed using resting-state fMRI and Coactivation Based Parcellation. *Hum. Brain Mapp.* 38 1659–1675. 10.1002/hbm.23488 28045222 PMC6867154

[B45] Wang L. Wei Q. Wang C. Xu J. Wang K. Tian Y. (2020). Altered functional connectivity patterns of insular subregions in major depressive disorder after electroconvulsive therapy. *Brain Imaging Behav.* 14 753–761. 10.1007/s11682-018-0013-z 30610527

[B46] Wang L. Yu L. Wu F. Wu H. Wang J. (2019). Altered whole brain functional connectivity pattern homogeneity in medication-free major depressive disorder. *J. Affect. Disord.* 253 18–25. 10.1016/j.jad.2019.04.040 31009844

[B47] Wang J. Ji Y. Li X. He Z. Wei Q. Bai T. (2020). Improved and residual functional abnormalities in major depressive disorder after electroconvulsive therapy. *Prog. Neuropsychopharmacol. Biol. Psychiatry* 100:109888. 10.1016/j.pnpbp.2020.109888 32061788

[B48] Wang J. Wei Q. Wang L. Zhang H. Bai T. Cheng L. (2018). Functional reorganization of intra- and internetwork connectivity in major depressive disorder after electroconvulsive therapy. *Hum. Brain Mapp.* 39 1403–1411. 10.1002/hbm.23928 29266749 PMC6866547

[B49] Wang J. Yang Y. Fan L. Xu J. Li C. Liu Y. (2015). Convergent functional architecture of the superior parietal lobule unraveled with multimodal neuroimaging approaches. *Hum. Brain Mapp.* 36 238–257. 10.1002/hbm.22626 25181023 PMC4268275

[B50] Wang Y. Zhang Y. Zhang J. Wang J. Xu J. Li J. (2018). Structural and functional abnormalities of the insular cortex in trigeminal neuralgia: a multimodal magnetic resonance imaging analysis. *Pain* 159 507–514. 10.1097/j.pain.0000000000001120 29200179

[B51] Woodworth D. C. Holly L. T. Mayer E. A. Salamon N. Ellingson B. M. (2019). Alterations in cortical thickness and subcortical volume are associated with neurological symptoms and neck pain in patients with cervical spondylosis. *Neurosurgery* 84 588–598. 10.1093/neuros/nyy066 29548020 PMC6500881

[B52] Woodworth D. C. Holly L. T. Salamon N. Ellingson B. M. (2018). Resting-state functional magnetic resonance imaging connectivity of the brain is associated with altered sensorimotor function in patients with cervical spondylosis. *World Neurosurg.* 119 e740–e749. 10.1016/j.wneu.2018.07.257 30092474 PMC6200587

[B53] Wu H. Sun H. Xu J. Wu Y. Wang C. Xiao J. (2016). Changed Hub and corresponding functional connectivity of subgenual anterior cingulate cortex in major depressive disorder. *Front. Neuroanat.* 10:120. 10.3389/fnana.2016.00120 28018183 PMC5159433

[B54] Xu J. Lyu H. Li T. Xu Z. Fu X. Jia F. (2019a). Delineating functional segregations of the human middle temporal gyrus with resting-state functional connectivity and coactivation patterns. *Hum. Brain Mapp.* 40 5159–5171. 10.1002/hbm.24763 31423713 PMC6865466

[B55] Xu J. Wang J. Fan L. Li H. Zhang W. Hu Q. (2015). Tractography-based Parcellation of the Human Middle Temporal Gyrus. *Sci. Rep.* 5:18883. 10.1038/srep18883 PMC4686935 26689815

[B56] Xu J. Wang C. Xu Z. Li T. Chen F. Chen K. (2019b). Specific functional connectivity patterns of middle temporal gyrus subregions in children and adults with autism spectrum disorder. *Autism Res.* 13 410–422. 10.1002/aur.2239 31729198

[B57] Yu C. X. Ji T. T. Song H. Li B. Han Q. Li L. (2017). Abnormality of spontaneous brain activities in patients with chronic neck and shoulder pain: a resting-state fMRI study. *J. Int. Med. Res.* 45 182–192. 10.1177/0300060516679345 28222620 PMC5536581

[B58] Zang Y. F. He Y. Zhu C. Z. Cao Q. J. Sui M. Q. Liang M. (2007). Altered baseline brain activity in children with ADHD revealed by resting-state functional MRI. *Brain Dev.* 29 83–91. 10.1016/j.braindev.2006.07.002 16919409

[B59] Zhang Y. Fan L. Zhang Y. Wang J. Zhu M. Zhang Y. (2014). Connectivity-based parcellation of the human posteromedial cortex. *Cereb. Cortex* 24 719–727. 10.1093/cercor/bhs353 23146967

[B60] Zou Q. H. Zhu C. Z. Yang Y. Zuo X. N. Long X. Y. Cao Q. J. (2008). An improved approach to detection of amplitude of low-frequency fluctuation (ALFF) for resting-state fMRI: fractional ALFF. *J. Neurosci. Methods* 172 137–141. 10.1016/j.jneumeth.2008.04.012 18501969 PMC3902859

